# Analysis of the Influencing Factors on the Extraction of Residual Oil through the Gel Foam Flooding of Underground Reservoirs in the Tahe Oilfield

**DOI:** 10.3390/gels9100804

**Published:** 2023-10-06

**Authors:** Chang-Ming Li, Ji-Rui Hou, Yu-Chen Wen, Tuo Liang

**Affiliations:** 1 Research Institute of Unconventional Petroleum Science and Technology, China University of Petroleum, Beijing 102249, China; lichangming0213@163.com; 2 Key Laboratory of Petroleum Engineering, China University of Petroleum, Beijing 102249, China; 3 College of Petroleum Engineering, Xi’an Shiyou University, Xi’an 710065, China; ta.liang@foxmail.com

**Keywords:** underground river, gel foam flooding, residual oil, enhanced oil recovery (EOR)

## Abstract

Fractured-vuggy reservoirs are mainly composed of three types: underground rivers, vugs, and fractured-vuggy structures. Based on the similarity criterion, a 3D model can truly reflect the characteristics of the multi-scale space of a fractured-vuggy reservoir, and it can reflect fluid flow laws in the formation. Water flooding, gas flooding, and gel foam flooding were carried out in the model sequentially. Based on gas flooding, the enhanced recovery ratio of gel foam flooding in the underground river was approximately 12%. By changing the injection rate, the average recovery ratio of nitrogen flooding was 6.84% higher than that of other injection rates at 5 mL/min, and that of gel foam flooding was 1.88% higher than that of other injection rates at 5 mL/min. The experimental results showed that the gel foam induced four oil displacement mechanisms, which selectively plugged high-permeability channels, controlled the mobility ratio, reduced oil-water interfacial tension, and changed the wettability of rock surfaces. With different injection-production methods, gel foam flooding can spread across two underground river channels. Two cases of nitrogen flooding affected one underground river channel and two underground river channels. By adjusting the injection rate, it was found that after nitrogen flooding, there were mainly four types of residual oil, and gel foam flooding mainly yielded three types of remaining oil. This study verified the influencing factors of extracting residual oil from an underground river and provides theoretical support for the subsequent application of gel foam flooding in underground rivers.

## 1. Introduction

Among the types of oil and gas reservoirs discovered worldwide, fractured-vuggy carbonate reservoirs play a crucial role [1]. Geological reserves account for 60% of the total reserves of conventional reservoirs, and their production occupies approximately 50% of the total production of conventional reservoirs [2,3]. Tahe oilfield, located in the Tarim Basin in western China, is a typical fractured-vuggy carbonate reservoir [4]. Based on the development characteristics of the fractured-vuggy system of the Ordovician carbonate reservoir in Tahe oilfield, the fractured-vuggy system can be categorized into three types: underground river system, vugs, and fractured-vuggy [5,6].

The subsequent energy in the reservoir is mainly supplied by water injection in different ways [7,8]. Many scholars have carried out extensive research on water flooding in fractured-vuggy reservoirs and put forward a variety of water flooding production models to improve the effect of water flooding [5,9,10]. For example, Khormali proposed an increase in displacement efficiency by injecting asphaltene inhibitors, which reduce the amount of asphaltene adsorbed on the rock surface [11]. Due to the strong heterogeneity of fractured-vuggy carbonate reservoirs, water flows tend to form dominant channels in fractures or vugs during water flooding, leading to water channeling and a poor water flooding effect [12,13]. Karimaie studied the oil recovery ratio of nitrogen gas and carbon dioxide in a fractured-vuggy carbonate reservoir and carried out physical simulation experiments to record the oil recovery ratio changes in the two gases with time. The research showed that gas flooding could displace residual oil after water flooding and reduce residual oil saturation [14]. Hui proved that nitrogen huff and puff replacement of remaining oil is a feasible EOR technology for fracture-vuggy carbonate reservoirs. [15]. 

The viscosity of injected gas is much lower than that of formation water and crude oil; the injected gas is prone to fingering during gas flooding. This fingering phenomenon leads to gas bypassing crude oil, reducing the sweep efficiency of gas flooding, and eventually causing gas channeling [16]. Foam can effectively increase gas phase viscosity and reduce gas fingering. Additionally, surfactants contained in the foam can effectively reduce the interfacial tension between oil and water; thus, foam has a good research prospect in the enhanced oil recovery (EOR) of fractured-vuggy carbonate reservoirs [17]. Li developed a visual-physical model based on a fractured-vuggy cross-section of the Tahe oilfield and carried out gas flooding and foam flooding experiments after water flooding. The experiments proved that foam can expand the sweep volume, emulsify, and carry residual oil to enhance the recovery ratio [18]. Yuan developed a 3D simulation model and carried out nitrogen gas flooding and foam flooding experiments. The research showed that foam flooding can effectively enhance the oil recovery of fractured-vuggy carbonate reservoirs, which is because the gas in the foam could start attic oil extraction. The surfactant could peel off the oil film and carry the oil droplets out through emulsification [9]. Yang performed experiments on nitrogen gas flooding and foam-assisted nitrogen gas flooding through a 2D visual model and found that the recovery factor of foam-assisted nitrogen gas flooding is 12% higher than that of nitrogen gas flooding. Foam can effectively control gas mobility and delay gas channeling [19]. Xu established a microscopic fractured-vuggy reservoir model and analyzed the flow characteristics and the enhanced oil recovery (EOR) mechanism of foam. The experiment showed that foam flooding can displace residual oil near the top of the vugs, high-speed foam flooding after low-speed foam flooding can better displace the oil in dead-end pores, and foam flooding can improve oil recovery from fractured vuggy reservoirs [20].

The migration behavior of foam in the experimental process is quite different from that in the actual reservoir. Especially for fractured-vuggy carbonate reservoirs, the mechanism of foam displacing the residual oil in vuggy is not clear, and the impact of various factors on enhanced oil recovery (EOR) needs to be further explored. Among the three fractured-vuggy structures in fractured-vuggy carbonate reservoirs, the underground river is a vuggy underground channel with the main characteristics of a river, representing the largest vuggy system. Research on it is of great significance for the subsequent development of fractured-vuggy carbonate reservoirs.

In this study, a 3D visual underground river fractured-vuggy reservoir model was designed by combining the actual data of oilfield production with visualized physical materials. We carried out experimental research on water flooding, nitrogen gas flooding, and gel foam flooding in the underground river model. By adjusting the injection speed and injection-production method of nitrogen gas flooding and gel foam flooding, the effects of enhanced oil recovery (EOR) under different factors were observed, the existing forms of residual oil in different stages were summarized, the mechanism of gel foam flooding in the start-up of the residual oil in the underground river model was analyzed, and the influencing factors of gel foam flooding on initiating residual oil were studied. This paper provides a valuable reference for gel foam flooding in the later stages of fractured-vuggy carbonate reservoirs.

## 2. Results and Discussion

### 2.1. Experimental Study of Gel Foam Flooding in the 3D Visual Underground River Physical Model

#### 2.1.1. Analysis of the Experimental Results of the 3D Visual Underground River Physical Model

1. Water flooding stage

**Figure 1:**
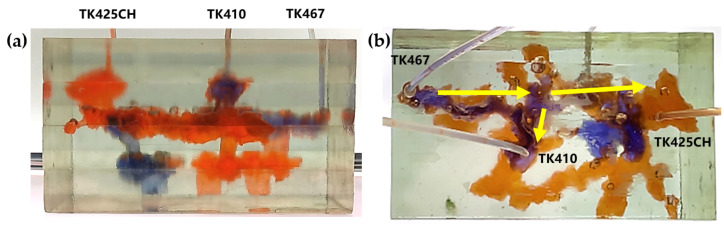
Water flooding stage of the 3D underground river model: (**a**) front view; (**b**) top view.

The 3D visual physical model was designed and developed based on the TK425CH unit and was a typical underground river reservoir physical model. The model mainly included two underground river channels, one located between TK425CH-TK467 and the other located in the TK410 well area. The bottoms of the TK410 and TK425 wells were located in the vuggy area, and the bottoms of the TK467 wells were located in the underground river channel area. In the water flooding stage, after water injection in the TK425CH well, the injected water first displaced the residual oil in the vuggy at the bottom of the well, then entered the underground river. The injected water migrated along the underground river channel in the direction of TK425CH-TK410 and then entered the vuggy at the bottom of well TK410. A water cone phenomenon occurred, resulting in the first water breakthrough in well TK410. The other underground river was not effectively swept by flooding, as shown in Figure 1, the yellow arrows in the figure indicate the flow direction of water flooding. The experimental results showed that the overall oil recovery of water flooding was low and there was still a large amount of residual oil at the bottom of the well; however, the water flooding exerted a strong displacement effect on the fractured-vuggy area around the injection well and the structure of the underground river.

2. Gas flooding stage

**Figure 2:**
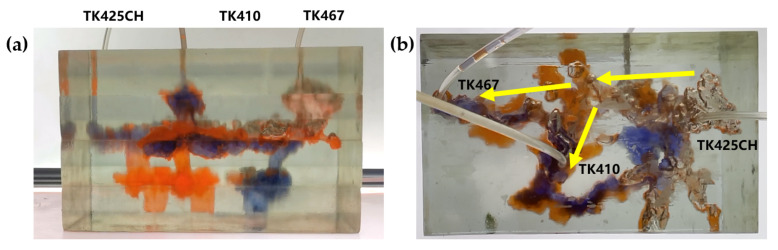
Gas flooding stage of the 3D underground river model: (**a**) front view; (**b**) top view.

Gas flooding experiments were conducted on the 3D visual underground river model of the TK425CH unit. The gas injection began in the TK425CH well; the injected gas entered the bottom of the TK425CH well, then replaced the residual oil in the surrounding vugs first. Subsequently, the gas entered the underground river and migrated along the underground river channel in the direction of TK425CH-TK467. As shown in Figure 2, yellow arrows in the figure indicate the flow direction of gas flooding, the effect of gas flooding was different from that of water flooding. The TK467 well on this channel was affected first, and then gas entered the vuggy at the bottom of the TK410 well along the underground river channel. The TK410 well was affected, and gas formed a gas cap in the vuggy to replace the attic oil at the top of the vuggy; however, the residual oil below the vuggy was not affected effectively. Finally, the dominant channel of gas channeling was formed in the direction of TK425CH-TK467, and the recovery ratio after gas flooding was 68.1%. As shown in Figure 1b and Figure 2b, both water flooding and gas flooding effectively affected only one underground river channel, and the main flow channels were different. Neither water nor gas flooding fully utilized the residual oil in the underground channel. After the formation of fluid flow-dominant channels in the underground river, a large amount of residual oil remained in the structure of the underground river and could not be affected. After the formation of dominant channels for fluid flow in the underground river, a large amount of residual oil existed in the underground river structure and could not be affected. Through comparison, it was found that the experimental phenomenon was consistent with the actual situation in the mine, which indicates that the experimental results have good accuracy and reference value.

3. Gel foam flooding stage

**Figure 3:**
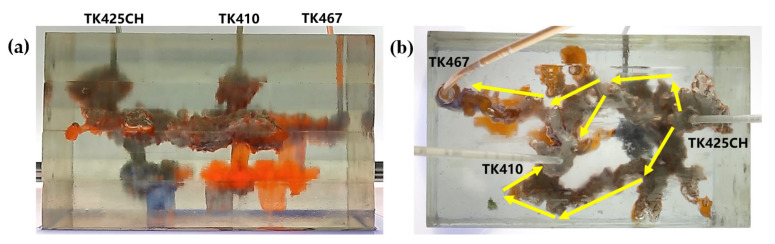
Gel foam flooding stage of the 3D underground river model: (**a**) front view; (**b**) top view.

The gel foam flooding experiment was carried out on the 3D visual underground river model in the TK425CH unit. Firstly, the gel foam was injected into the TK425CH well. As shown in Figure 3, water flooding and gas flooding only formed a single flow dominant channel, and the subsequent gel foam flooding could effectively start two underground river channels at the same time, the yellow arrows in the figure indicate the flow direction of gel foam flooding. After gel foam injection in the TK425CH well, the injected gel foam exhibited a good displacement effect on the bottom vugs. Then, after the gel foam entered the underground channel, the channeling flow after water and gas flooding was plugged, and the subsequent fluid flow direction was adjusted. Two different underground channels were started, which had a good displacement effect on the upper and lower parts of the underground river. Finally, the recovery ratio of gel foam flooding was 79.6%. This proved that gel foam can control the direction and rate of fluid in the displacement of fractured-vuggy reservoirs and can prevent the fluid from migrating along the water, inhibiting gas channeling. Finally, gel foam initiated contact with the fractured-vuggy structure that could not be reached by water flooding and gas flooding and carried out uniform displacement in multiple directions with good displacement efficiency. 

**Figure 4:**
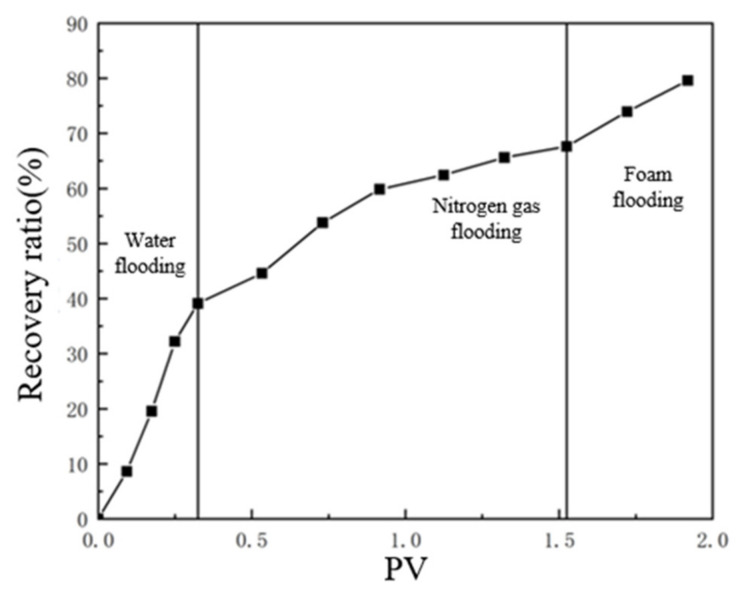
Relationship curve between the recovery ratio and PV in a displacement experiment of a 3D underground river model.

After water flooding had formed a dominant channel in the TK467 well, the recovery ratio of the TK425CH unit 3D visual underground river model was 39.7%. Subsequently, gas injection was carried out in the TK425CH well, and after gas channeling, the final recovery ratio reached 68.2%, with an increase of 28.5%. In the actual production process, the average water flooding utilization degree in Tahe District 4 was 37.43%, and the gas flooding utilization degree was 30.3%, which is consistent with the experimental results. As shown in Figure 4, 0.4 PV gel foam was injected into the underground river unit model; the final recovery ratio was 79.6%, and the recovery ratio was increased by 11.4%. 

#### 2.1.2. Impact of the Injection-Production Method on the Displacement Effect of the 3D Visual Underground River Physical Model

1. Nitrogen gas flooding at different well locations

**Figure 5:**
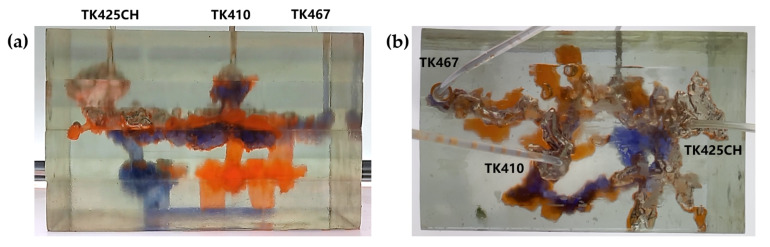
Gas injection in the TK425CH well of the 3D underground river model: (**a**) front view; (**b**) top view.

**Figure 6:**
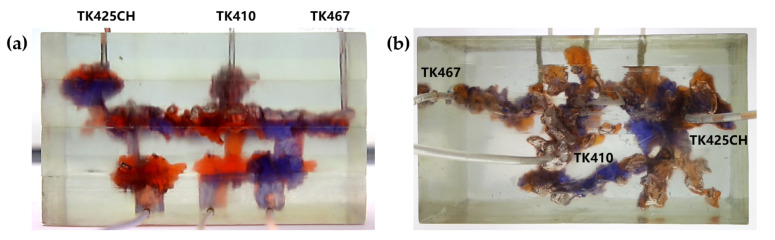
Gas injection in the TK410 well of the 3D visual physical model: (**a**) front view; (**b**) top view.

Based on the TK425CH unit 3D visual underground river model, nitrogen gas flooding experiments were conducted at different well locations to study the nitrogen gas flooding displacement effect at different well locations in the fractured-vuggy reservoir. Experiments showed that when TK425CH was a gas injection well, the injected gas first gathered at the top of the vugs at the bottom of the TK425CH well. Most of the residual oil in the fractured-vuggy structure near the TK425CH well was replaced. Then, the injected gas entered the underground river channel. Nitrogen gas mainly migrated forward along the TK425CH-TK467 channel, as shown in Figure 5. When the TK410 well was a gas injection well, the injected gas first replaced most of the attic oil in the TK425CH well-bottom vugs. After entering the underground river channel, nitrogen gas mainly migrated in two directions: TK410-TK467 and TK410-TK425CH. After arriving at the TK425CH well, nitrogen gas flooding had a certain displacement effect on the residual oil in the vugs at the bottom of the well, as shown in Figure 6. Compared with TK425CH, during the gas injection process in the TK410 well, more underground river channels were started, the nitrogen gas swept volume was wider, and the recovery ratio was higher. After water flooding, the final recovery ratio for gas flooding was 71.3%. However, after water flooding in TK425CH, the final recovery ratio for gas flooding was 67.9%.

2. Gel foam flooding at different well locations

**Figure 7:**
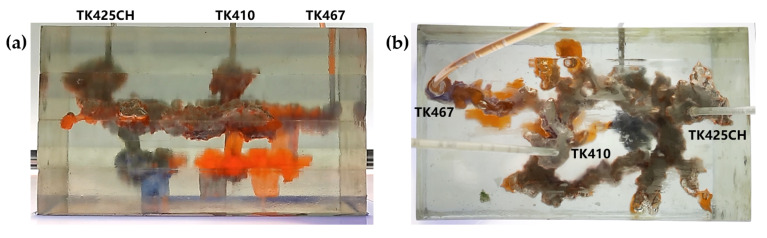
Gel foam injection in the TK425CH well of the 3D underground river model: (**a**) front view; (**b**) top view.

**Figure 8:**
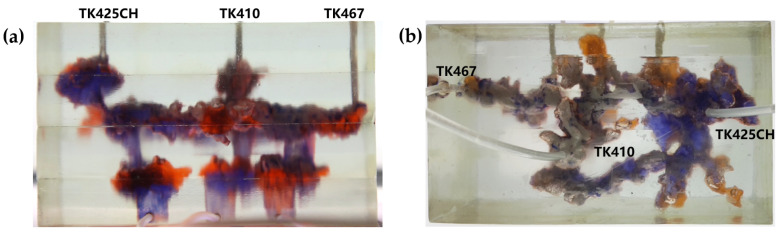
Gel foam injection in the TK410 well of the 3D underground river model: (**a**) front view; (**b**) top view.

Based on the TK425CH well unit 3D visual underground river model, gel foam flooding experiments at different well locations were carried out to study the gel foam flooding displacement effect at different well locations in fractured-vuggy reservoirs. The experimental results are shown in Figure 7 and Figure 8. After gel foam was injected into the TK425CH well, gel foam first entered the vugs at the bottom of the TK425CH well, effectively displacing residual oil in the vugs at the bottom of the TK425CH well. After entering the underground channel, the gel foam migrated along the two directions of TK425CH-TK467 and TK425CH-TK410. Gel foam exhibited a good displacement effect on the underground channel and the fractured-vuggy structure connected by the channel. However, the displacement effect of gel foam on the underground river channel in the TK410-TK467 direction was poor, and the final recovery ratio of gel foam flooding was 79.6%. After gel foam was injected into well TK410, gel foam effectively displaced the residual oil in the vuggy at the bottom of well TK410. After entering the underground channel, gel foam effectively started two underground river channels, TK410-TK467 and TK410-TK425CH.

**Figure 9:**
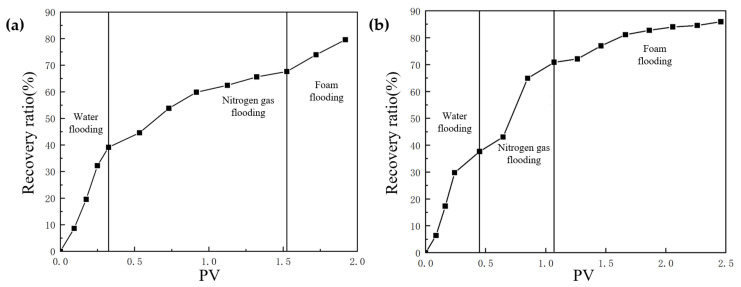
Relationship curve between the recovery ratio and PV in a displacement experiment of the 3D underground river model: (**a**) TK425CH well; (**b**) TK410 well.

The displacement effect of the TK410-TK467 channel was better than that of gel foam injection in the TK425CH well, which could effectively displace the corner residual oil of the underground river tributaries. The gel foam flooding final recovery ratio was 86.1%, 6.5% higher than that of gel foam injection in the TK425CH well, as shown in Figure 9.

#### 2.1.3. Impact of the Injection Rate on the Displacement Effect of the 3D Visual Underground River Physical Model

1. Nitrogen flooding gas with different injection rates

**Figure 10:**
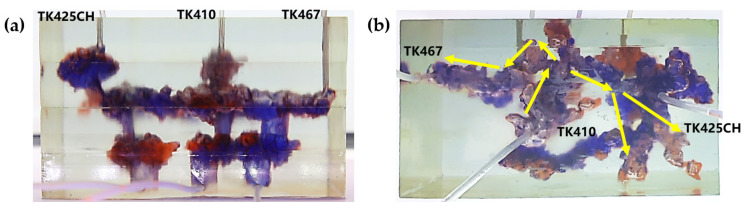
Gas injection in the TK410 well of the 3D underground river model at 5 mL/min: (**a**) front view; (**b**) top view.

**Figure 11:**
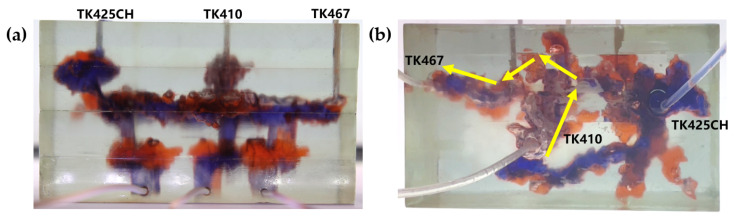
Gas injection in the TK410 well of the 3D underground river model at 10 mL/min: (**a**) front view; (**b**) top view.

**Figure 12:**
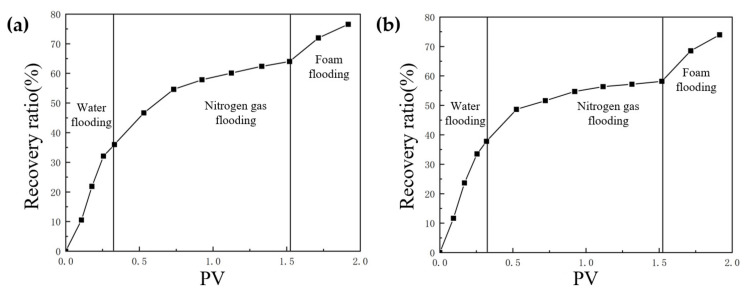
Relationship curve between the recovery ratio and PV in the displacement experiment of the 3D underground river model: (**a**) gas rate of 5 mL/min; (**b**) gas rate of 10 mL/min.

Based on the TK425CH unit 3D underground river visual model, nitrogen gas flooding experiments with different injection rates were carried out to study the nitrogen gas flooding displacement effect at different rates in fractured-vuggy reservoirs. Through experiments, it was found that when TK410 was a gas injection well with a nitrogen gas injection rate of 5 mL/min, the injected gas first migrated to the bottom of the production well through the vugs located in the upper part connecting the fracture. The gas gathered at the top of the vugs at the bottom of TK410, and the flow component of the injected gas continued to increase. Then, the residual oil in the surrounding vugs was quickly replaced, and after entering the underground river, it migrated along the underground river channel in the direction of TK410-TK467. The TK467 well on this channel was affected first. After the attic oil around the TK410 well was completely displaced, gas entered the vugs at the bottom of the TK425CH well along the underground channel. The injected gas formed a gas cap in the vugs to replace the attic oil at the top of the vugs. The TK410 well was also affected; however, the residual oil in the lower part of the vugs was not effectively affected. Finally, a dominant gas channel was formed in the TK410-TK425CH direction. The experimental results are shown in Figure 10, the yellow arrows in the figure indicate the flow direction of gas flooding. When TK410 was a gas injection well and the nitrogen gas injection rate was 10 mL/min, the gas channeling component of the injected gas increased. The displacement component of the injected gas decreased along the model channel, and the injected gas migrated along the underground river channel in the direction of TK410-TK467, resulting in oil production from well TK467. The experimental results are shown in Figure 11, the yellow arrows in the figure indicate the flow direction of gas flooding. Compared with the two injection rates of nitrogen gas, when the injection rate of nitrogen gas was 5 mL/min, the dominant channel in the TK410-TK467 direction was formed, gas could continue to migrate in the direction of TK410-TK425CH, and residual oil around the TK425CH well was displaced. Compared with the nitrogen gas injection rate of 10 mL/min, more underground river channels were displaced, the sweep effect was better, and the recovery ratio was higher. The final recovery ratio after gas flooding was 66.4%, as shown in Figure 12. 

**Figure 13:**
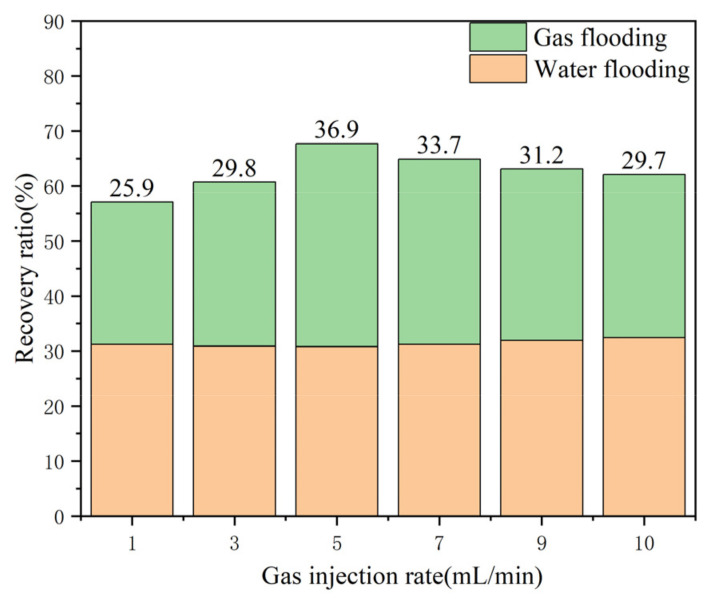
Diagram of the nitrogen flooding recovery ratio with different injection rates.

It can be concluded that in the process of gas flooding after water flooding, it is necessary to ensure that the injection rate of nitrogen gas is not too fast, and too high a gas injection rate cannot fully enable nitrogen gas flooding in the EOR. To prevent gas channeling and ensure that the displaced volume is maximized, a reasonable injection rate can achieve the optimal oil recovery effect. As shown in Figure 13, a consistent water flooding rate was ensured, the optimal nitrogen injection rate was 5 mL/min, and the average recovery rate was increased by 6.84%. Therefore, the optimal injection rate should be controlled at approximately 5 mL/min.

2. Gel foam flooding with different injection rates

**Figure 14:**
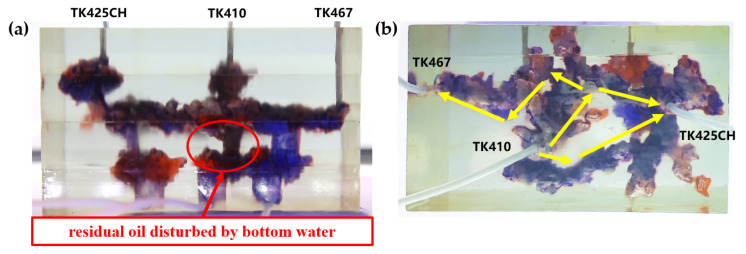
Gel foam injection in the TK410 well of the 3D underground river model at 5 mL/min: (**a**) front view; (**b**) top view.

**Figure 15:**
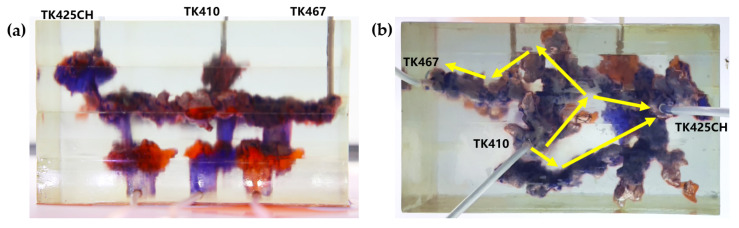
Gel foam injection in the TK410 well of the 3D underground river model at 10 mL/min: (**a**) front view; (**b**) top view.

**Figure 16:**
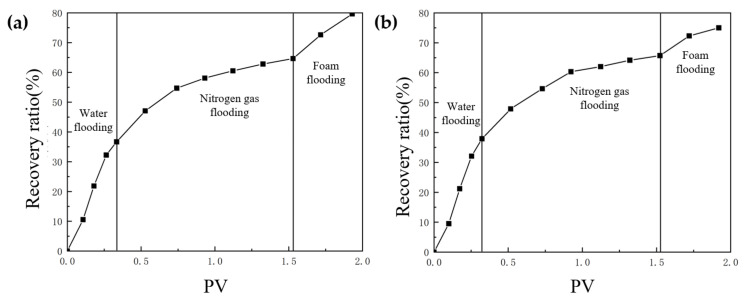
Relationship curve between the recovery ratio and PV in the displacement experiment of the 3D underground river model: (**a**) gel foam rate with 5 mL/min; (**b**) gel foam rate with 10 mL/min.

Based on the TK425CH unit 3D underground river visual model, gel foam flooding experiments with different injection rates were carried out to study the gel foam flooding displacement effects at different rates in fractured-vuggy reservoirs. Through experiments, it was found that when TK410 was a gel foam injection well with a gel foam injection rate of 5 mL/min, the gel foam effectively displaced the residual oil in the vugs at the bottom of the TK410 well. After entering the underground channel, gel foam migrated along TK410-TK467 and TK410-TK425CH, demonstrating a good displacement effect on the underground channel and the connected fractured-vuggy structure, as shown in Figure 14, the yellow arrows in the figure indicate the flow direction of gel foam flooding. When TK410 was an injection well and the injection rate of gel foam was 10 mL/min, the gel foam effectively displaced the residual oil in the vugs at the bottom of the TK410 well and migrated along the two directions of TK410-TK467 and TK410-TK425CH; however, the sweet effect on the TK410-TK425CH underground river channel was poor, as shown in Figure 15, the yellow arrows in the figure indicate the flow direction of gel foam flooding. Compared with the two gel foam injection rates, when the gel foam injection rate was 5 mL/min, the sweep effect on the underground river channel was better, which could effectively displace some corner residual oil on the underground river tributaries. Gel foam also had a certain effect on the residual oil disturbed by bottom water below the vugs; the final recovery ratio after gel foam flooding was 79.6%, as shown in Figure 16.

**Figure 17:**
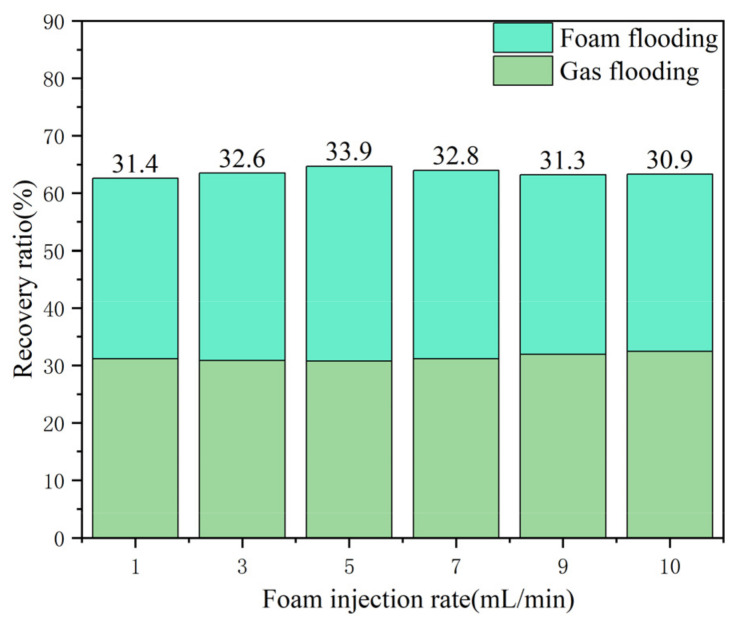
Graph of the gel foam flooding recovery ratio with different injection rates.

Due to the bursting and regeneration of gel foam in the process of formation migration, when the gel foam is injected into vugs, the gas will exist in the form of a dispersed phase. When the injection rate of gel foam was too high, the injection pressure rose rapidly. The gas in the gel foam changed from the dispersed phase to the continuous phase, forcing the gas phase to flow separately from the liquid phase. The flow capacity of the gas phase was higher than that of the liquid phase; therefore, the phenomenon of channeling was prone to occur, leading to a decrease in the oil recovery ratio. According to the experimental results, gel foam flooding had a strong displacement effect after gas flooding; however, the selection of gel foam injection rate was also crucial. As shown in Figure 17, when consistent water flooding and nitrogen gas rates were ensured, the optimal gel foam injection rate was 5 mL/min, and the average recovery rate increased by 6.84%. Therefore, the optimal injection rate should be controlled at approximately 5 mL/min. 

#### 2.1.4. The Distribution of Residual Oil in Different Displacement Stages of the 3D Visual Underground River Physical Model

**Figure 18:**
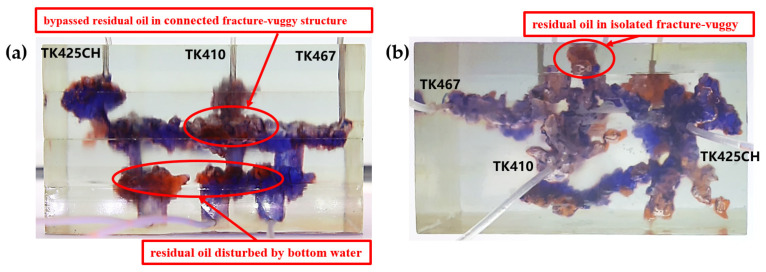
The distribution of residual oil after gas flooding in the 3D underground river model (gas rate of 5 mL/min): (**a**) front view; (**b**) top view.

**Figure 19:**
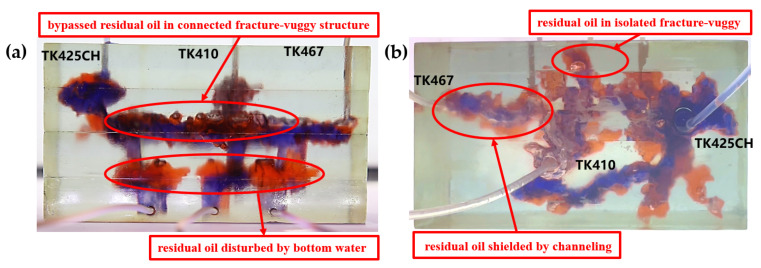
The distribution of residual oil after gas flooding in the 3D underground river model (gas rate of 10 mL/min): (**a**) front view; (**b**) top view.

Residual oil in the visual underground river physical model displacement experiment can be divided into the following types: residual oil disturbed by bottom water, bypassed residual oil in the connected fractured-vuggy structure, residual oil in the isolated fractured-vuggy structure, residual oil shielded by channeling and residual oil at the blind end vug. Residual oil in isolated fractured-vuggy structures mainly existed in small reservoirs, one of the main reservoirs of residual oils after gas flooding. Taking the top view of Figure 18 and Figure 19 as an example, there is an isolated fractured-vuggy structure above the middle of the image. Due to the effect of gas gravity differentiation, gas flooding could not displace residual oil in the fractured cavity, and the residual oil existed in the isolated fractured-vuggy structure Due to the presence of bottom water, a portion of the fractured-vuggy structure was submerged. The residual oil disturbed by bottom water was the residual oil submerged in the fractured-vuggy structure, which is hardly affected by conventional displacement methods. Taking the effect of gas flooding swept in the front view of Figure 19 as an example, gas flooding only affected the underground river channel above the model, and the residual oil surrounded by bottom water below the model was not affected. This residual oil is disturbed by bottom water. When the displacement medium was injected into the fractured-vuggy structure, the flow channel was formed. In the underground river channel, some residual oil was still adsorbed on the rock surface after the displacement medium was displaced; the residual oil that had not been swept was the bypassed residual oil in the connected fractured-vuggy structure. Taking the effect of gas flooding swept in the front view of Figure 19 as an example, an underground river channel was displaced after gas flooding. After gas flooding, there is still some residual oil on the wall around the underground river channel on the left side of the model; this residual oil was bypassed in the connected fractured-vuggy structure. When the displacement medium was injected into the fractured-vuggy structure, the channeling channel was formed, and the residual flow channels could not be effectively affected, forming residual oil shielded by channeling. When the displacement medium was injected into the fracture-cave structure, a channeling flow channel was formed, which could not effectively influence another flow channel, and residual oil in the other flow channel was shielded by channeling. In the top view of Figure 19, for example, an underground river channel was used after gas flooding. Another underground river channel on the left of the top view was not affected, and the residual oil in this underground river channel was shielded by channeling. The residual oil at the blind end of the vug was mainly caused by the complex fractured-vuggy structure, which generated some unconnected pores containing residual oil. Conventional displacement media could not replace the oil in the blind end and corner structures; residual oil existed at the blind end.

1. The types of residual oil after gas flooding

Figure 18 shows the distribution diagram of residual oil after nitrogen gas displacement at 5 mL/min in a 3D visual underground river reservoir. After gas flooding in the 3D visual underground river fractured-vuggy reservoir, there were three main types of residual oil: a small portion of bypassed residual oil in the connected fractured-vuggy structure, residual oil in the isolated fractured-vuggy structure, and a small portion of residual oil disturbed by bottom water.

Figure 19 shows the distribution diagram of residual oil after nitrogen gas displacement at 10 mL/min in a 3D visual underground river reservoir. After gas flooding in the 3D visual underground river fractured-vuggy reservoir, there were four main types of residual oil: bypassed residual oil in the connected fractured-vuggy structure, residual oil in the isolated fractured-vuggy structure, residual oil shielded by channeling, and residual oil disturbed by bottom water.

2. The types of residual oil after gel foam flooding

**Figure 20:**
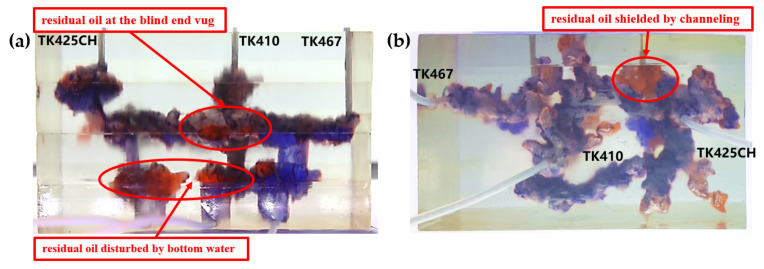
The distribution of residual oil after gel foam flooding in the 3D underground river model (gel foam rate of 5 mL/min): (**a**) front view; (**b**) top view.

Figure 20 shows the distribution diagram of residual oil after gel foam displacement at 5 mL/min in a 3D visual underground river reservoir. After gel foam flooding in the 3D visual underground river fractured-vuggy reservoir, there were three types of residual oil: residual oil shielded by channeling, residual oil at the blind end vug, and a small portion of residual oil disturbed by bottom water. 

**Figure 21:**
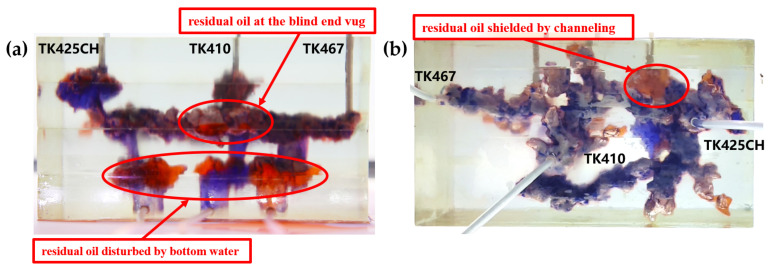
The distribution of residual oil after gel foam flooding in the 3D underground river model (gel foam rate of 10 mL/min): (**a**) front view; (**b**) top view.

Figure 21 shows the distribution diagram of residual oil after gel foam displacement at 10 mL/min in a 3D visual underground river reservoir. After gel foam flooding in the 3D visual underground river fractured-vuggy reservoir, there were three main types of residual oil: residual oil shielded by channeling, residual oil at the blind end vug, and residual oil disturbed by bottom water.

In the experiment, the residual oil displacement scenarios were observed by simulating different injection rates during nitrogen flooding and foam flooding in fractured-vuggy reservoirs. The experiment showed that different nitrogen gas injection rates cause differences in the utilization of the remaining oil. Compared with the injection rate of 10 mL/min, when nitrogen gas was injected at 5 mL/min, it could effectively displace the residual oil shielded by channeling, and the utilization degree of residual oil disturbed by bottom water and bypassed residual oil in the connected fractured-vuggy structure was also relatively high. Compared with the injection speed of 10 mL/min for gel foam, when gel foam was injected at 5 mL/min, the utilization of residual oil disturbed by bottom water was also relatively high. 

### 2.2. Mechanical Mechanism of Gel Foam Leaving Residual Oil in the Underground River System

#### 2.2.1. Wettability Alternation

**Figure 22:**
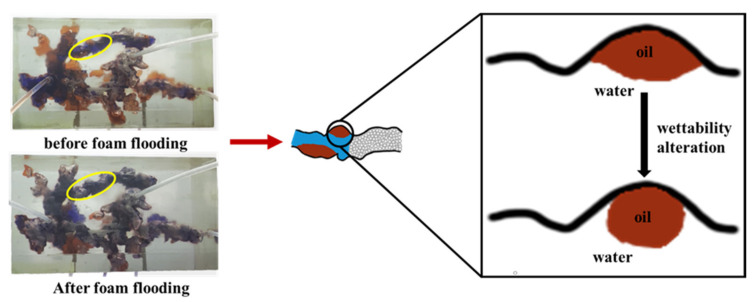
Schematic diagram of rock surface wettability alterations.

After the gel foam was injected into the model, as seen from the top view, the gel foam migrated forward in the form of pistons through irregular channels. The surfactant molecules in the gel foam were able to change the rock surface from lipophilic to hydrophilic, changing the wettability of the rock surface and increasing the wettability angle, θ, as shown in Figure 22. In Figure 22, the schematic diagram of the yellow circle is shown in the middle picture, while the oil displacement process of the gel foam at the yellow circle is shown in the right picture. The formula for calculating adhesion work was obtained from Equation (1). When the wetting angle increased, it can be seen from the formula that the adhesion work decreased. When the adhesion work is less than the cohesion value of crude oil, S > 0, the oil droplets contract on the rock surface. Oil droplets are easily displaced by the gel foam and eventually migrate out of the gel foam.

(1) $$W_{adhesion}=\sigma_{S/O}+\sigma_{W/O}-\sigma_{W/S}=\sigma_{W/O}(COS\theta +1)$$

(2) $$S=W_{cohesion}-W_{adhesion}$$

where $W_{adhesion}$ is the adhesion work, $\sigma_{S/O}$ is the interfacial tension between the rock surface and oil, $\sigma_{W/O}$ is the interfacial tension between oil and water, $\sigma_{W/S}$ is the interfacial tension between the rock surface and water, θ is the contact angle, $W_{cohesion}$ is the cohesion work, and S is the spreading coefficient.

#### 2.2.2. Selective Plugging Effect

**Figure 23:**
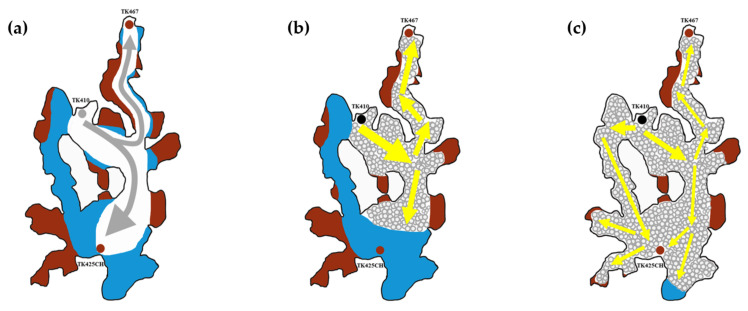
Schematic diagram of gel foam selective plugging: (**a**) late stage of gas flooding; (**b**) initial stage of gel foam flooding; and (**c**) late stage of gel foam flooding.

As seen from the top view, after gel foam was injected into the model from well TK410, it first gathered in the vugs at the bottom of the well. The flow resistance of the underground river between well TK410 and well TK467 was low; therefore, gel foam first entered the large underground river, displacing most of the residual oil in the big channel from the produced well. Oil saturation in the large underground river was reduced, and the stability of the gel foam was enhanced. With the continuous injection of gel foam, gel foam gathered in the large underground river and increased its flow resistance. When gel foam gathered to a certain extent, the flow resistance of the large underground river gradually increased, inducing changes in the flow direction of the injection of gel foam. Subsequently, gel foam entered the small underground river between well TK410 and well TK425CH and finally displaced the residual oil shielded by channeling and residual oil at the blind end vug, as shown in Figure 23, the grey arrows in the figure indicate the flow direction of gas flooding, and the yellow arrows in the figure indicate the flow direction of gel foam flooding.

#### 2.2.3. Mobility Control Ability

After the gel foam was injected from the injection well, it first gathered near the well, and then the gel foam slowly migrated along the large channel. The movement of the gel foam trapped the gas, and the rich liquid film in the gel foam restricted the flow of the gas. In the process of gel foam migration in the underground river, new bubbles were constantly generated, which reduced the relative permeability of gas, increased the apparent gas viscosity, and delayed gas channeling.

(3) $$M=\frac{\lambda_{D}}{\lambda_{d}}=\frac{K_{D}\mu_{d}}{{K_{d}\mu }_{D}}$$

where M is the mobility ratio, $\lambda_{D}$ is the mobility of the displacement phase, $\lambda_{d}$ is the mobility of the displaced phase, $K_{D}$ is the permeability of the displacement phase, $K_{d}$ is the permeability of the displaced phase, $\mu_{d}$ is the viscosity of the displaced phase, and $\mu_{dD}$ is the viscosity of the displacement phase.

#### 2.2.4. Gravity Differentiation

**Figure 24:**
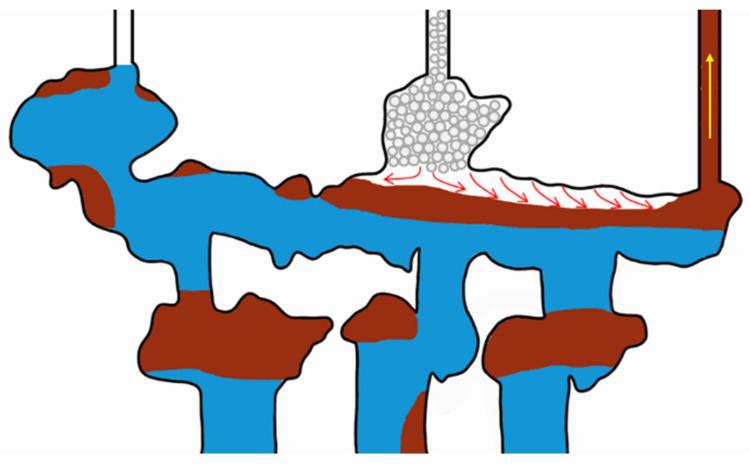
Schematic diagram of gel foam gravity differentiation.

Gel foam exhibits the characteristics of defoaming when encountering oil and stabilizing when encountering water. When the gel foam came into contact with oil in the large channel, the gel foam became unstable and burst. Due to gravity differentiation, the gas in the gel foam gathered above the channel after the gel foam burst, forming a gas cap. As more and more gel foam burst, gas gathered and squeezed the residual oil below, thus forcing the residual oil out of the outlet, as shown in Figure 24, the red arrows in the figure indicate the flow direction of gas, and the yellow arrows in the figure indicate the flow direction of oil. 

## 3. Conclusions

In this study, a 3D visual underground river physical model of a fractured-vuggy reservoir was established based on similarity criteria, and experimental simulations of gel foam flooding were carried out. This study verified the feasibility of gel foam flooding in the EOR of fractured-vuggy carbonate reservoirs.

1. Regarding the impact of injection and production methods on oil recovery in underground river fractured-vuggy reservoirs, the results showed that in the process of oil displacement, actual reservoir conditions should be considered. For the well group with good connectivity in the fractured-vuggy reservoir, the effect of gas flooding on EOR is poor, and the structure of the fractured-vuggy reservoir contains a large amount of residual oil. Gel foam flooding can be carried out for wells connecting multiple vugs, and the recovery ratio of the well group can be improved by combining multiple displacement methods. Based on gas flooding, the enhanced recovery ratio of gel foam flooding in the underground river was approximately 12%.
2. The impact of injection speed on oil recovery was studied. Gel foam flooding can spread across two underground river channels; two cases of nitrogen flooding affected one underground river channel and two underground river channels with different injection rates. In underground rivers, increasing the injection rate sufficiently can improve oil recovery by increasing the injection pressure. When the gas injection rate reaches 10 mL/min, the difference in oil and gas mobility quickly causes gas channeling, shortening the gas injection time and yielding a lower final recovery ratio than that for a gas injection rate of 5 mL/min. The average recovery ratio of nitrogen flooding was 6.84% higher than that of other injection rates at 5 mL/min. In the process of gel foam injection, gel foam plugged the high-permeability channel and delayed gas channeling to a certain extent. The average recovery ratio of gel foam flooding was 1.88% higher than that of other injection rates at 5 mL/min. When the injection rate of gel foam is too high, the gas will break through the gel foam to form a channeling channel, which will lead to a decline in the oil displacement effect. Therefore, whether gas flooding or gel foam flooding, a reasonable injection rate can achieve the comprehensive oil displacement effect of delaying gas channeling and increasing swept volume.
3. Through oil displacement experiments at different stages on 3D visual models, it was found that after nitrogen flooding, there were mainly four types of residual oil, and gel foam flooding mainly yielded three types of remaining oil. Research showed that, compared with 10 mL/min nitrogen gas displacement, 5 mL/min nitrogen gas displacement can displace residual oil shielded by channeling, most of the bypassed residual oil in connected fractured-vuggy structures, and most of the residual oil disturbed by bottom water. The effect of 5 mL/min nitrogen gas displacement was better. Compared with 10 mL/min gel foam flooding, 5 mL/min gel foam flooding can displace most of the residual oil disturbed by bottom water, and 5 mL/min gel foam flooding had a better displacement effect.
4. The experiments demonstrated that gel foam exerts an oil displacement mechanism that changes the wettability of rock surfaces, a selective plugging effect, mobility control ability, and gravity differentiation in fractured-vuggy carbonate reservoirs.

## 4. Materials and Methods

### 4.1. Similarity Design of the Physical Model

The flow laws of fluid in a fractured-vuggy carbonate reservoir are complicated. To simulate the geological condition and reservoir space of oilfields as accurately as possible, the physical model should reflect the characteristics of multi-scale fractured-vuggy reservoir space and reflect the coexistence of multiple flows. The theoretical basis of physical simulation is the similarity theory; the similarity criterion guides the fabrication of physical models. There are two common methods for deriving similarity criteria, one of which is dimension analysis, and the other is equation analysis. To ensure the suitability and applicability of the derived results, dimension analysis was chosen to deduce the similarity criterion.

According to the similarity theory of physical simulation, to achieve similarity between the model and the prototype, the physical model used in the experiment should meet the three conditions of geometric, kinematic, and dynamic similarity principles in design. Due to the complex heterogeneity of fractured-vuggy reservoirs, it is impossible to achieve complete similarity criteria. Therefore, in the process of experimental analysis, it is necessary to focus on the key points, select the main similarity criteria, and ignore the secondary similarity criteria; as such, the laboratory physical simulation experiment can reflect the actual reservoir to a certain extent. This experiment focuses on the selection of kinematic and dynamic similarity criteria to study the mechanism. An oilfield in Tahe was selected as the research object, the geological data were extracted, and the similarity design was defined by combining the parameters of fracture size, tortuosity, vuggy size, etc. A model similar to the actual reservoir was produced by scaling down. By selecting different parameters as references and combining them with the flow law of fluid in the actual reservoir, the similarity criterion formula was calculated using the dimension rule. 

The correlation data of the actual reservoir parameters and physical models are included in the similarity criterion formula for calculation. The similarity coefficient is defined as the ratio of the physical model parameters to the actual reservoir parameters. The similarity index is defined as the ratio of the calculated results obtained by bringing two parameters into the formula. The similarity index represents the similarity between the physical model and the actual reservoir. The closer the similarity index is to 1, the higher the similarity between the physical model and the actual reservoir. Therefore, the similarity index was set to 1 to calculate the parameters of the physical model. The main similarity criteria of physical simulation are shown in Table 1, and the parameter comparison and similarity coefficient between the reservoir prototype and physical model are shown in Table 2. 

In the experiment, kerosene and liquid paraffin were used to prepare simulated oil with the same viscosity as crude oil under actual reservoir conditions, and the simulated oil was dyed with Sudan red. The simulated formation water was prepared according to the salinity and ion content of reservoir formation water, which ensured that the fluid medium used in the physical simulation experiment was the same as the actual fluid. The experimental fluid was closer to the actual fluid flow law, ensuring the reliability of the physical simulation results.

### 4.2. Physical Model Design and Fabrication

**Figure 25:**
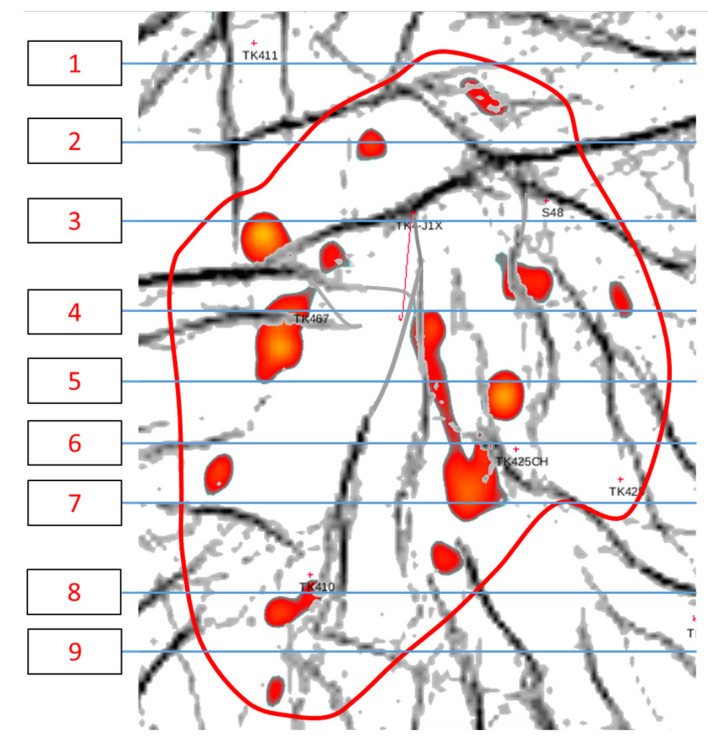
Schematic drawing of the cross-section of the TK425CH well group. The TK425CH well group was divided into nine equal parts. The numbers 1–9 represent geological drawing of longitudinal sections at different locations in the well group.

**Figure 26:**
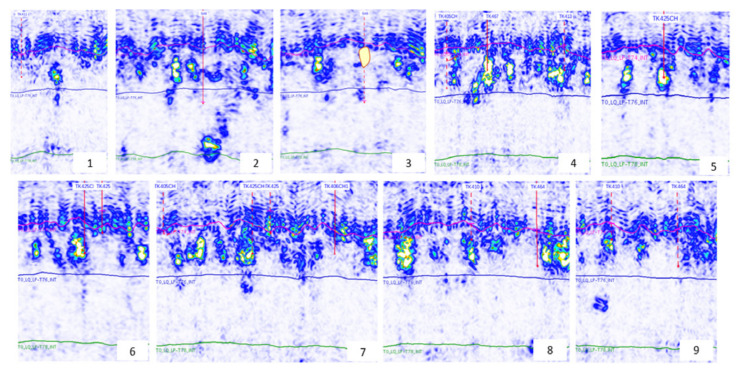
Geological drawing of longitudinal sections of the TK425CH well group. The numbers 1–9 represent different geological drawings of longitudinal sections. The location number of each longitudinal section in Figure 25 corresponds to the number of its corresponding geological drawing in Figure 26.

**Figure 27:**
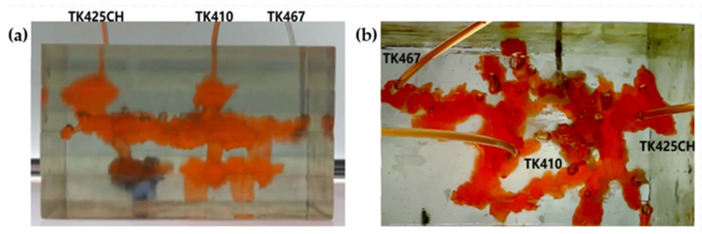
Schematic diagram of TK425CH well group locations in a 3D underground river model: (**a**) front view; (**b**) top view.

To study the flow law and oil displacement effect of gel foam in different well locations and injection rates, as well as the residual oil production in different stages in fractured-vuggy reservoirs, we designed a 3D visual fractured-vuggy reservoir model. By taking the TK425CH well group unit of the Tahe fractured-vuggy reservoir as the research object, based on the similarity criteria, we extracted the 3D geological carving data volume and obtained the overall geological characteristics. As shown in Figure 25 and Figure 26, nine cross-sections of the fractured-vuggy body of the well group were equidistantly intercepted to obtain the cross-section of multiple simulated oil layers. The obtained fractured-vuggy structure data were imported into 3D simulation software. Each simulated fractured-vuggy unit was arranged in the same section according to the cross-section of each oil layer. After optimizing the design, each simulated oil layer was produced using a 3D printer. Then, the simulated oil layers were superimposed and bonded according to the overall geological characteristics, and it was ensured that the adjacent joints of fractured-vuggy units were interconnected, resulting in a 3D visual fractured-vuggy reservoir model. The fractures, or vugs, in the 3D visual fractured-vuggy reservoir model, were formed using transparent materials. The organic glass material used was polymethyl methacrylate, which has good temperature resistance and chemical stability. The oil’s wettability was consistent with the wettability of the core. The above characteristics can reduce experimental errors. We added the location of injection-production well groups to the physical profile map, as shown in Figure 27. The 3D visual fractured-vuggy reservoir physical model of well group TK425CH was 15 cm × 8 cm × 8 cm. 

### 4.3. Experimental Materials and Equipment

The experimental simulated oil was configured using liquid paraffin and kerosene in proportion. The viscosity of the simulated oil was 23.9 mPa·s, and the density of the simulated oil was 0.821 g/mL at 25 °C. To facilitate observation under visual conditions, the simulated oil was dyed red with Sudan III. The experimental water was configured based on the actual formation water ionic composition of Tahe oilfield, with a viscosity of 1 mPa·s, a density of 0.98 g/mL, and a mineralization degree of about 250,000 mg/L. To facilitate observation under visual conditions, the experimental water was dyed blue with methylene blue dye. The composition and specific content of ions in water are shown in Table 3. The foam used in the experiment was gel foam, and α-starch nanogel particles were added to the foam, which yielded the foam with highly stable properties during the migration process in the fractured-vuggy reservoir. The chemical reagents used to prepare the highly stable gel foam included the foaming agent (SS-163, Qingdao Changxing High-tech Development Co., Ltd., Qingdao, China), sodium dodecyl sulfate, α-modified starch, acrylamide, N, N′-methylenebisacrylamide, and potassium persulfate. The preparation method was detailed in our previous study [21]. The gas used in the experiment was industrial nitrogen (99% purity). 

**Figure 28:**
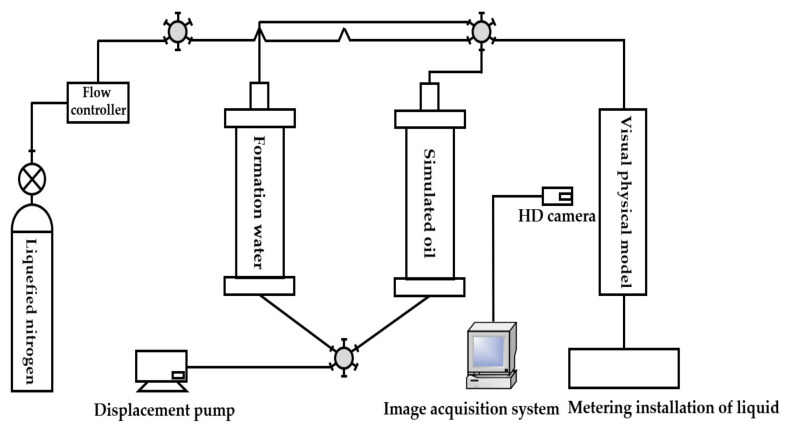
Flow chart of a physical simulation experiment of gas displacement.

**Figure 29:**
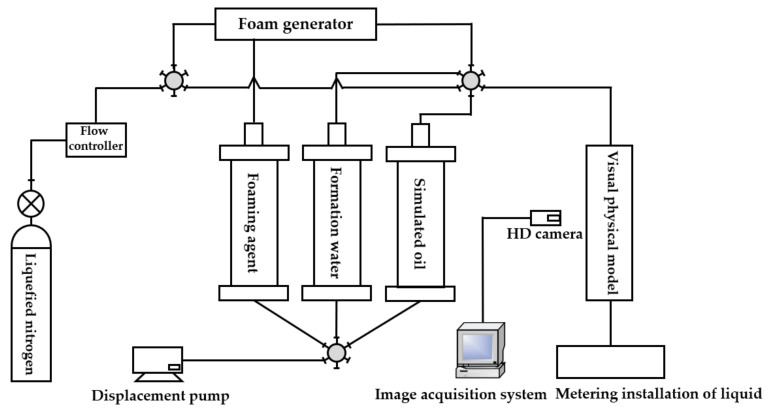
Flow chart of a physical simulation experiment of gel foam displacement.

The gel foam flooding physical simulation experiment equipment mainly consisted of three parts: a visual physical model system, an experimental control system, and an image and data acquisition system, as shown in Figure 28 and Figure 29. The experimental control system was composed of an injection pump (the working pressure was 0~30 MPa and the flow rate was 0.001~20 mL/min), a nitrogen gas bottle, a gas flow controller, a six-way valve, a gel foam generator, and intermediate containers containing gel foam liquid, simulated oil, and simulated formation water. The image and data acquisition system included a metering installation of liquid and a high-definition camera with a maximum resolution of 1080P. The model used in the experiment was the 3D visual underground river fractured-vuggy reservoir model.

### 4.4. Experimental Methods and Procedures

Under normal temperature and pressure (25 °C and 0.1 MPa, respectively), water flooding, gas flooding, and gel foam flooding physical simulation experiments were carried out successively in the 3D visual underground river physical mode. By observing the 3D visual physical model, the effect of EOR in the fractured-vuggy reservoir can be observed, and the remaining oil displacement law of fractured-vuggy structures can be analyzed. The specific experimental process is as follows:

a. The 3D visual underground river fractured-vuggy reservoir physical mode was saturated with oil.
b. Water flooding was carried out in an injection TK425CH well with a water injection rate of 2 mL/min (the production wells were TK410 and TK467). Water injection was stopped when the water cut at the production channel reached 98% or the water channeling was complete.
c. Gas flooding was carried out in the TK425CH well with a gas injection rate of 5 mL/min. The gas injection was stopped until gas channeling occurred at the production channel.
d. Gel foam flooding was carried out in a TK425CH well with an injection rate of 2 mL/min. Gel foam flooding was stopped until no oil was produced at the production channel.
e. The experimental process was recorded using an HD camera. The experimental schemes are shown in Table 4.

## Figures and Tables

**Table 1:** The main similarity criteria of physical simulation.

Similarity Conditions	Similarity Criteria	Physical Significance	Similarity Index
Dynamic similarity	F_G_ = ΔP/(ρ_o_gd)	Ratio of the injected pressure to gravity	1.01–1.04
	Re = ρul/μ	Ratio of the moving inertial force to the viscous force	1
Kinematic similarity	F_Q_ = Q/(r^2^ u)	Ratio of the recovery volume to the injection volume	1.01–1.04
Characteristic similarity	π_2_ = ξ	Coordination number	1
	π_3_ = η	Filling degree	1

**Table 2:** Parameter comparison and similarity coefficient between the reservoir prototype and the physical model.

Parameters	Reservoir Model	Physical Model	Similarity Coefficient
Cavity diameter (d)/cm	500–2500	3–12	166.667–200
Pressure difference (Δp)/kPa	2000–14,000	10–60	200–233.333
Viscosity of crude oil (μ)/(mPa·s)	19.7–28.5	65	0.3038–0.4377
Density of crude oil (ρo)/(g·cm^−3^)	0.92	0.8	1.15
Gravity acceleration (g)/(m·s^−2^)	9.8	9.8	1
Seepage velocity (u)/(m·s^−1^)	0.0147–0.147	0.007–0.049	2.103–2.993
Injection velocity (Q)/(m^3^·d^−1^)	20–60	0.006–0.015	3334–4000
Well diameter (r)/mm	120	3	40
Fracture aperture(B)/mm	0.5–5	0.2–4.5	0.25–1.11
Filling degree	0–100%	0–100%	1

**Table 3:** Formation of water ion composition.

Total Salinity/mg·L^−1^	Ionic Concentration/mg·L^−1^					
	Na^+^	Ca^2+^	Mg^2+^	Cl^−^	HCO_3_^−^	SO_4_^2−^
253,225	82,023	14,849	905	154,748	418	282

**Table 4:** Physical experiment schemes.

	Injection Well	Water Flooding	Nitrogen Gas Flooding	Foam Flooding
1	TK425CH	2 mL/min	5 mL/min	2 mL/min
	TK410			
	TK467			
2	TK425CH	2 mL/min		
	TK410		5 mL/min	2 mL/min
	TK467			
3	TK425CH			
	TK410		5 mL/min	1, 3, 5, 7, 9, 10 mL/min
	TK467	5 mL/min		
4	TK425CH			
	TK410		1, 3, 5, 7, 9, 10 mL/min	10 mL/min
	TK467	5 mL/min		

## Data Availability

Data are available from the corresponding author upon reasonable request.
